# Potential Effects of Bisphenol A on the Heart and Coronary Artery of Adult Male Rats and the Possible Role of L-Carnitine

**DOI:** 10.1155/2022/7760594

**Published:** 2022-12-26

**Authors:** Mohamed Moharram Badawy, Mohsen M. Elsherbiny, Gehad Elsaid Elshopakey, Asmaa Ezat Elsayyad, Mohammad Abd-El-Same'e El-Kattan, Mohamed G. Hamama, Fatemah H. Aldariweesh, Alaa Fehaid

**Affiliations:** ^1^Department of Forensic Medicine and Clinical Toxicology, Faculty of Medicine, Mansoura University, Mansoura, Egypt; ^2^Department of Forensic Medicine and Clinical Toxicology, Faculty of Medicine, Delta University for Science and Technology, Gamasa, El Dakahleya, Egypt; ^3^Department of Health Informatics, Faculty of Public Health and Tropical Medicine, Jazan University, Jazan, Saudi Arabia; ^4^Department of Clinical Pathology, Faculty of Veterinary Medicine, Mansoura University, Mansoura, Egypt; ^5^Department of Pharmacology, Faculty of Veterinary Medicine, Mansoura University, Mansoura, Egypt; ^6^Anatomy Department, Faculty of Medicine, Tanta University, Tanta, Egypt; ^7^Environmental Health Department, Public Health Department, Ministry of Health, Kuwait City, Kuwait; ^8^Department of Forensic Medicine and Toxicology, Faculty of Veterinary Medicine, Mansoura University, Mansoura, Egypt

## Abstract

Bisphenol A (BPA) is an environmental toxin utilized for the production of polycarbonate plastics and epoxy resins. Due to BPA's extensive production and environmental contamination, human exposure is unavoidable. The effects of low-dose of BPA on various body tissues and organs remain controversial. Our study investigated the potential of BPA to induce biochemical, histopathological, and immunohistochemical changes in the coronary artery and myocardium and the potential protective role of L-carnitine (LC). 24 adult Wistar albino male rats were divided equally into a control group, a BPA-treated group (40 mg/kg/d, by gavage for 4 weeks), and a BPA plus LC-treated group (received 40 mg/kg/d of BPA and 300 mg/kg/d of LC, by gavage for 4 weeks). BPA-exposed rats demonstrated structural anomalies in the coronary artery tissue including vacuolation of cells in the media and detachment of the endothelium of the intima. Congestion of blood vessels and infiltration by polynuclear cells were observed in the myocardium. There was an enhanced collagen deposition in both tissues indicating fibrosis. Immunohistochemical changes included enhanced eNOS and caspase-3 expression in the coronary artery and myocardium indicating vascular disease and apoptosis, respectively. Oxidative damage was evident in the coronary artery and the myocardium of BPA-treated rats, which was indicated by the reduced level of glutathione (GSH) and elevated malondydehyde (MDA) levels. The coadministration of LC significantly improved BPA-induced structural alterations and oxidative stress. In conclusion, BPA could potentially cause pathologic changes and oxidative damage in the coronary artery and myocardium, which could be improved by LC coadministration.

## 1. Introduction

Bisphenol A is one of the greatest volumes of chemicals produced globally, and more than one hundred tons are released into the environment every year [1]. It is used in the manufacturing of polycarbonate plastic products, which include water bottles, sports equipment, CDs, and DVDs. BPA is also utilized to line water pipes, and food cans, and in the production of thermal paper [2]. Due to its widespread use, human exposure to BPA is universal. More than a decade ago, measurable BPA concentrations were detected in 93% of urine samples from humans [3]. Since there is continuous exposure to BPA, this chemical has been detected in almost all humans studied [4].

The health hazards related to BPA are mostly because of the partial polymerization leaving some unbound monomer BPA molecules in the product. Such monomers become released into food or beverages with time, particularly due to the effects of heat, acidic, or basic conditions [5]. It was stated that humans are exposed to BPA predominantly in their diet; however, there is evidence that exposure could also happen through inhalation of household dust or particles released during the BPA's industrial production [6].

Bisphenol A acts through several mechanisms. More specifically, it interacts with multiple receptors such as estrogen, aryl hydrocarbon, androgen, thyroid hormone, and glucocorticoid receptors. BPA binds to classical nuclear estrogen receptors, classical and nonclassical membrane-bound ERs, and G protein-coupled receptors 30 [7]. Moreover, BPA can cause oxidative stress in different body tissues as reported in several studies [8, 9]. In addition, BPA can act through epigenetic mechanisms and alterations in genomic methylation, including genes involved in immunological function, transport activity, metabolism, and chromosome X [10].

Though the endocrine-disrupting effects of BPA have been thoroughly evaluated, there is still some uncertainty regarding its potential to harm the health. Much of such uncertainty is because of the controversies that surround the design and interpretation of results from hypothesis-driven BPA research [11].

Being an endocrine disruptor, BPA can trigger several disorders, including developmental and reproductive system abnormalities, impaired brain and neurologic functions, malignancy, cardiovascular disease (CVD), diabetes, early puberty, obesity, and immune dysregulation [12].

Cardiovascular diseases are the leading cause of mortality globally. Research has found that oxidative damage has a significant role in the development of coronary artery disease (CAD) [13]. A systematic review has demonstrated a correlation between increased BPA and the enhanced risk of CVD, obesity, diabetes, insulin resistance, and hypertension in humans [14].

Experimentally, it was shown that low BPA doses could affect estrogen signaling in cultured rodent cardiomyocytes [15]. Also, exposure to oral BPA (50 mg over 8 weeks) caused morphologic and structural alterations in the rat myocardial tissue, including vacuolation of myocytes, focal loss of myofibrils, and mitochondrial distortion [16]. However, the impact of low-dose BPA on rat myocardial tissue remains elusive. Kim and coworkers demonstrated that BPA (50 *μ*g/kg/d) accelerated atherosclerosis progression in a genetic mouse model prone to endothelial dysfunction and vasculitis [17].

BPA exposure was also associated with atherosclerosis progression in the coronary artery and aorta of rabbits [18], and other reports have shown a relationship of BPA with blood pressure in mice [19], and electrical contraction of rat heart extract [20].

In addition, Aboul Ezz and colleagues administered BPA to adult male rats by oral route for 6–10 weeks (25 or 10 mg/kg/d) and assessed oxidative damage in the cardiac tissue. They reported enhanced lipid peroxidation, low GSH, and catalase levels. BPA was also associated with a reduction in nitric oxide, which may result in vasoconstriction and reduced blood supply to the cardiac tissue. There was also reduced acetylcholinesterase activity among the BPA-treated rats, which might cause a decrease in heart rate and contractility [21].

The cardiotoxic effects of BPA could be triggered through its action on estrogenic receptors, modification of cardiac Ca^2+^-handling protein expression, ion channel inhibition/activation, oxidative damage, production of free radicals, and genome/transcriptome modifications [22, 23].

LC is a nonprotein amino acid, which is formed from lysine and methionine amino acids. It promotes the *β*-oxidation of fatty acids and has a role in the metabolism of branched-chain amino acids and the stabilization of cell membranes [24]. Also, several studies have shown that LC acts as a free radical scavenger and thus provides protection for the antioxidant enzymes against oxidative stress [25, 26]. Compared to the wealth of data linking BPA to reproductive disorders, diabetes, and obesity, few studies have focused on the relationship of BPA with CVDs and in particular coronary artery pathology. Therefore, this work was conducted to assess the biochemical and histopathological alterations mediated by BPA in the myocardium and coronary artery of adult rats and to assess the effect of coadministration of LC against the BPA-induced alterations.

## 2. Martial and Methods

### 2.1. Ethical Consideration

In the current study, we followed the ethical guidelines according to the ethical norms approved by the Medical Research Ethics Committee, Mansoura University (Code No.: R/96).

### 2.2. Chemicals

BPA and LC were purchased from Sigma–Aldrich Inc. The required doses underwent dissolution in corn oil and were given to experimental animals by oral gavage.

### 2.3. Animals and Experimental Design

The study was performed on 24 male adult Wistar albino rats weighing about 200–250 g. Rats were housed and maintained under standard environmental conditions at a temperature of 22°C ± 2°C, 12 h of light/dark cycle, 41%–55% relative humidity, and provided with food and water throughout the experimental period. All animal work was carried out in accordance with the guidelines for the use of animals in research established by Mansoura University, Egypt. Also, the animals underwent handling according to the Guide for the Care and Use of Laboratory Animals [27].

The study duration was 4 weeks. The rats were randomly divided into three groups (8 rats each) as follows: Group 1: untreated rats and served as controls (received corn oil), Group 2 (BPA 40 mg/kg/d): rats were treated with 40 mg/kg/d BPA dissolved in corn oil and given by oral gavage. This dose was chosen following the Chapel Hill BPA expert panel consensus statement, which defined the “low BPA dose” dose below the LOAEL (50 mg/kg/d) in animal models [28], and Group 3 (BPA 40 mg/kg/d + LC 300 mg/kg/d): rats received 40 mg/kg/d BPA along with the administration of LC 300 mg/kg/d [29], dissolved in corn oil and given by gavage by oral gavage.

At the end of the experimental work, rats were intraperitoneally injected with pentobarbital (50 mg/kg). Their hearts were then dissected. The left ventricles were cut perpendicular to the long axis into rings of 1   –2 mm width and utilized for the measurement of tissue GSH and MDA, and histopathological and immunohistochemical examination.

### 2.4. Histopathological Examination

The myocardial and coronary artery tissues of the sacrificed rats were dissected immediately, and part of it was fixed in 10% neutral buffered formalin for histopathologic examination. The paraffin-embedded sections were prepared to be 5 *μ*m thicknesses, and deparaffinized using 100% xylene, followed by rehydration with 100% and then 70% ethyl alcohol. Transverse sections underwent staining with hematoxylin and eosin (HE) for visualization of general tissue morphology and Masson trichrome to detect fibrotic areas. Stained sections were examined under light microscopy [30, 31].

### 2.5. Immunohistochemistry

For the immunohistochemical study, sections were cut at 3 *μ*m thickness. They were collected on poly-L-lysine coated slides, dried in a thermostat at 37°C for 24 h to achieve an appropriate adhesion of the biologic material to the surface of the slide, and then stained with the appropriate antibodies (anti-eNOS and anticaspase-3). Immunohistochemistry examination by eNOS was performed according to Felaco et al. [32], while examination by caspase-3 was performed according to Hamed et al. [33].

### 2.6. Biochemical Tests

The myocardial and coronary artery specimens from the sacrificed rats were homogenized in phosphate buffer solution (0.01 M sodium phosphate buffer, pH 7.4, containing 0.14 M NaCl) at 1 ml volume/g tissue wet weight ratio of 4 : 1. Homogenates underwent centrifugation at 13,000 × *g* for 20 minutes, and the resultant supernatant was used for oxidative activity analysis. GSH and MDA concentrations were measured in the tissue homogenates utilizing commercially available kits.

### 2.7. Statistical Analysis

The data were analyzed using IBM SPSS Statistics for Windows, v 22.0. Armonk, NY: IBM Corp. Qualitative data were presented as numbers and percentages. Quantitative data were presented as means and standard deviations for parametric data following testing normality by the Kolmogorov –Smirnov test. The significance of a result was judged at the (0.05) level. The one-way ANOVA test was utilized to compare more than 2 independent groups with the post hoc Tukey test to detect pair-wise comparison.

## 3. Results

There were no reported mortalities among the experimental animals. In each group, the same area of each section was selected for hematoxylin and eosin, Masson trichome, eNOS, and caspase-3 stainings. The current study revealed the following findings.

### 3.1. Histopathology

#### 3.1.1. Hematoxylin and Eosin

**Figure 1:**
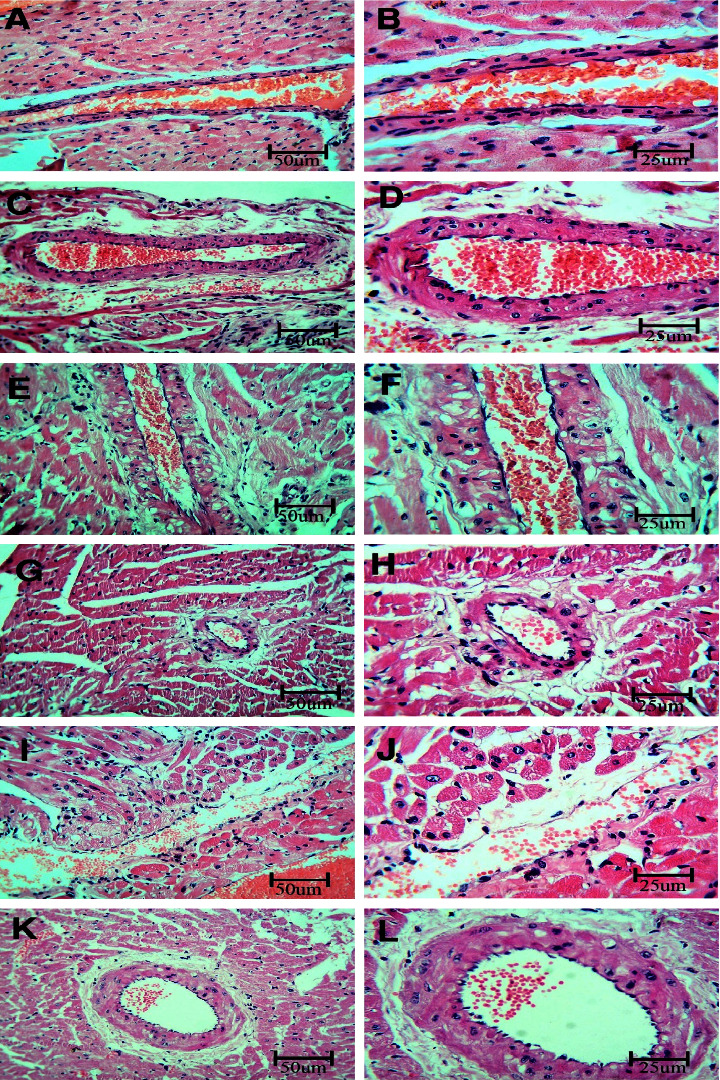
Histopathological section in the coronary artery of a rat stained with hematoxylin and eosin. a, b, c, and d are the control group; e, f, g, and h are the BPA group; and i, j, k, and l are the BPA + LC group. The L.S. sections (a and b) of the coronary artery of the control group and c and d are the T.S. section showing the normal structure of the artery with thick media rich in acidophilic elastic fibers and regular endothelium. The sections of the BPA group show weak acidophility and vacuolation of cells in the media and detachment of the endothelium of the intima of the artery. This picture is not marked in the BPA + LC group (HE stain; A, C, E, G, I, and K X 200; B, D, F, H, J, and L X 400).

**Figure 2:**
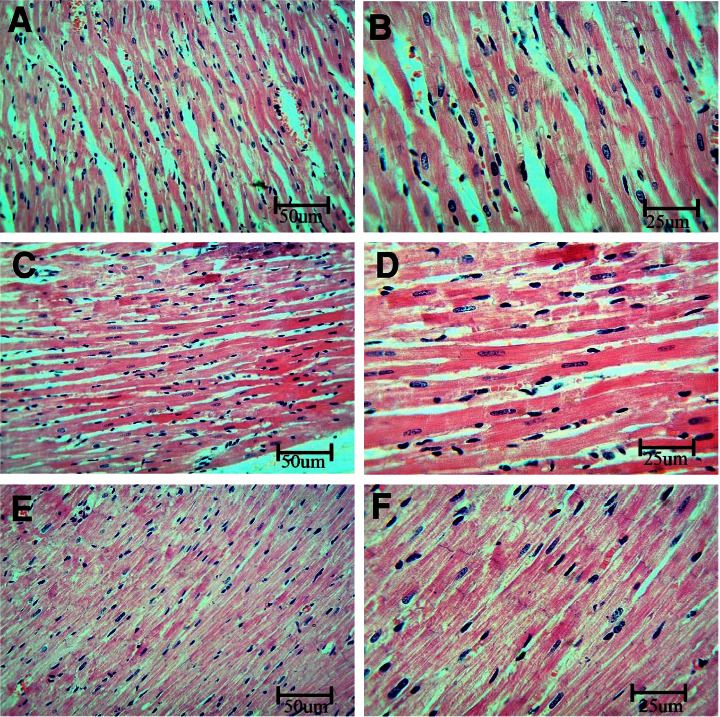
Histopathological section in the heart of rat stained with hematoxylin and eosin. a and b are the control group, c and d are the BPA group, and e and f are the BPA LC group. The sections of the BPA show congested blood vessels and infiltration by polynuclear cells. This picture is not marked in the BPA + LC group (HE stain; A, C, E, G, I, and K X 200; B, D, F, H, J, and L X 400).

To investigate BPA's effects on the coronary artery, sections were stained using HE and examined using a light microscope. Unlike the control rats, which demonstrated the normal histological structure of the artery, sections from the BPA group show vacuolation of cells in the media and detachment of the endothelium of the intima of the artery. In contrast, sections taken from rats that received the BPA + LC demonstrated a near-normal structure to control rats (Figure 1). Regarding the myocardium, the control group demonstrated branching and anastomosing muscle fibers. Cardiomyocytes show central oval nuclei. Capillaries and fibroblasts were observed in the connective tissue endomysium in between muscle fibers. On the other hand, the hearts of BPA-treated rats showed congested blood vessels and infiltration by polynuclear cells in comparison to control rats. Coadministration of LC improved these BPA-induced changes (Figure 2).

#### 3.1.2. Masson Trichrome Stain

**Figure 3:**
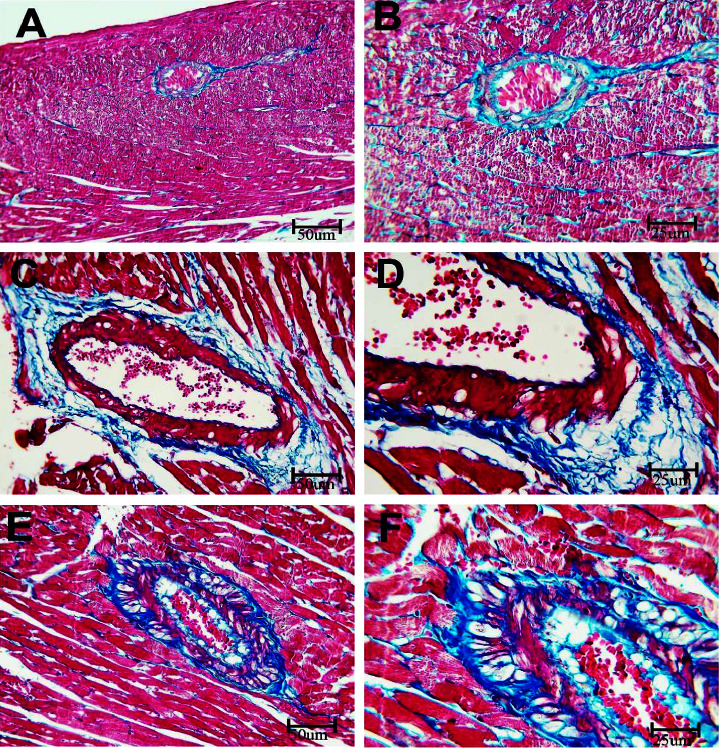
Histopathological section in the coronary of rat stained with Masson trichrome stain. a and b are the control group, c and d are the BPA group, and e and f are the BPA + LC group. The coronary artery of the control group shows minimal bluish-stained collagen fiber in the wall of the artery. The expression of the color is notably increased in the BPA group and reduced in the BPA + LC group (Masson trichrome stain; A, C, and E X 200; B, D, and F X 400).

**Figure 4:**
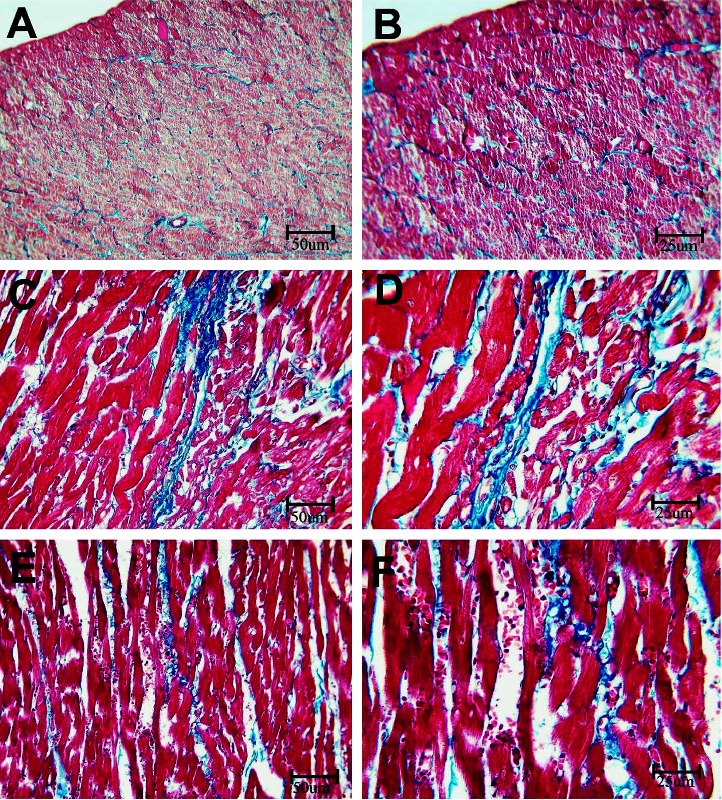
Histopathological section in the heart of rat stained with Masson trichrome stain. a and b is the control group, c and d are the BPA group, and e and f are the BPA + LC group. The myocardium of the control group shows minimal bluish-stained collagen fibers. The expression of the color is notably increased in the BPA group and reduced in the BPA + LC group (Masson trichrome stain; A, C, and E X 200, B, D, and F X 400).

**Figure 5:**
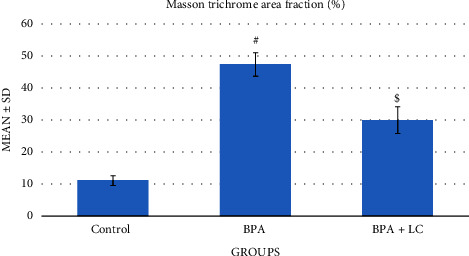
Graph demonstrating the quantitative analysis of interstitial collagen fiber area percentage in the heart. Data are expressed as means ± SDs. ^#^: significantly different from the control group, while ^$^significantly different from the BPA group (*P* < 0.001).

The Masson Trichrome stain stains the collagen-rich fibrotic areas in a bluish color. As demonstrated in Figure 3, the expression of the bluish color is notably enhanced in rats receiving BPA in comparison with rats in the BPA + LC group, which demonstrated reduced bluish color expression, indicating that BPA resulted in significant fibrosis in the wall of the coronary artery (*P* < 0.001) (Table 1). On the other hand, the Masson trichrome stain of the hearts demonstrated that BPA significantly enhanced the collagen fiber content in the myocardium in comparison with the control group (*P* < 0.001). However, LC coadministration was associated with a significant reduction in collagen deposition between muscle fibers (Figure 4) (*P* < 0.001). Moreover, as shown in Figure 5, the quantitative analysis of the interstitial collagen fiber area percentage in the heart revealed that BPA increased the fibrous tissue content compared to the control group (47.34 ± 3.60% versus 11.11 ± 1.54%). However, coadministration of LC and BPA caused a significant reduction in collagen deposition in comparison with the BPA-treated rats (30.02 ± 4.09% versus 47.34 ± 3.60%) (*P* < 0.001).

### 3.2. Immunohistochemistry

#### 3.2.1. Caspase-3

**Figure 6:**
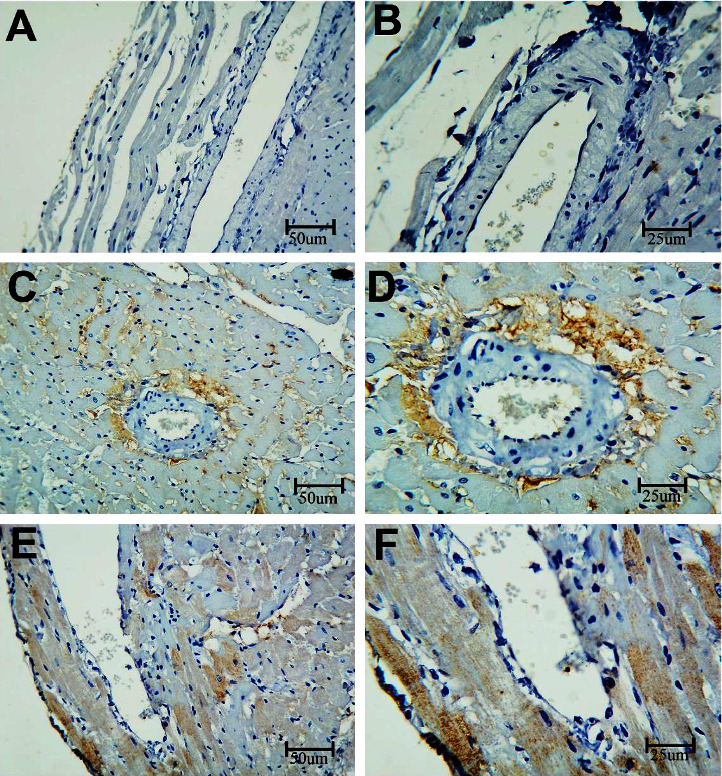
Histopathological section of the coronary artery of the rat stained with the caspase-3 immune stain. a and b are the control group, c and d are the BPA group, and e and f are the BPA + LC group. The coronary artery of the control group shows a minimal expression of the dye in the wall of the artery. The expression of the color is notably increased in the BPA group and reduced in the PBA + LC group (caspase-3 immune stain; A, C, and E X 200; B, D, and F X 400).

**Figure 7:**
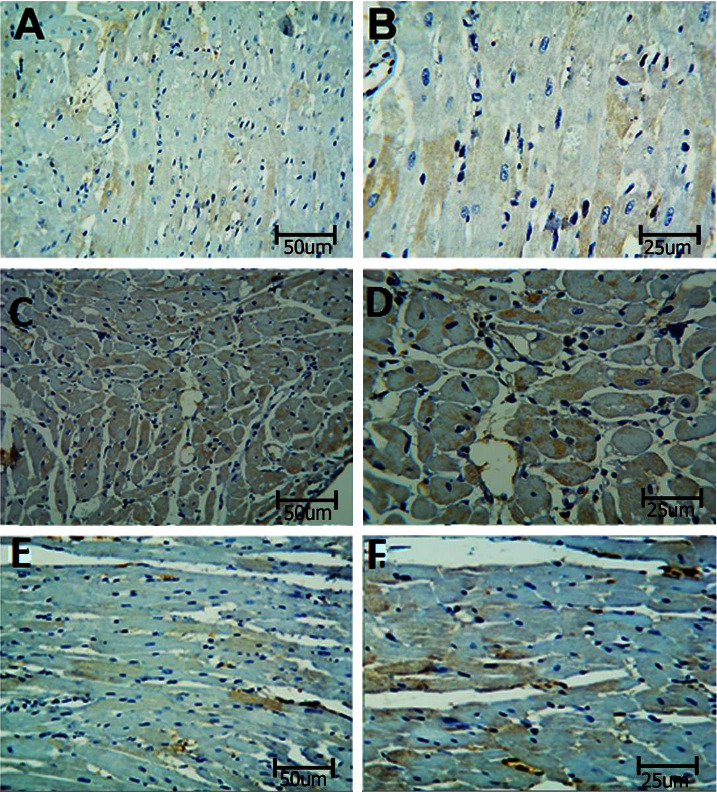
Histopathological section in the heart of rat stained with caspase-3 immune stain. a and b are the control group, c and d are the BPA group, and e and f are the BPA + LC group. The cardiac muscle of the control group shows a minimal expression of the dye in the wall of the artery. The expression of the color is notably increased in the BPA group and reduced in the BPA + LC group (caspase-3 immune stain; A, C, and E X 200, B, D, and F X 400).

**Figure 8:**
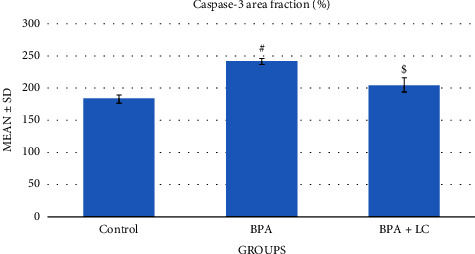
Graph demonstrating the quantitative analysis of caspase-3 expression area percentage of the heart. Data are expressed as means ± SDs. ^#^: significantly different from the control group, while ^$^significantly different from the BPA group (*P* < 0.001).

To measure the apoptosis in the coronary artery, sections were stained with the caspase-3 immune stain (Figure 6). BPA-exposed rats showed an increased expression of caspase-3 as compared with control rats. In contrast, the BPA + LC group demonstrated a decrease in caspase-3 expression in comparison to BPA-treated rats (*P* < 0.001) (Table 1). The apoptosis in the heart was also assessed in this study. Heart sections of BPA-exposed rats showed an increased caspase-3 expression (evidenced by excess brownish staining) as compared with control rats (*P* < 0.001). In contrast, the BPA + LC group showed a decrease in caspase-3 expression in comparison to the BPA group (Figure 7) (*P* < 0.001). The quantitative analysis of caspase-3 expression area percentage of the heart demonstrated that BPA enhanced caspase-3 expression in comparison with control rats (240.69 ± 4.84% versus 182.92 ± 6.44%) and that coadministration of LC and BPA was associated with a significant reduction in caspase-3 expression in comparison with the BPA group (204.47 ± 10.59% versus 240.69 ± 4.84%) (*P* < 0.001) (Figure 8).

#### 3.2.2. eNOS

**Figure 9:**
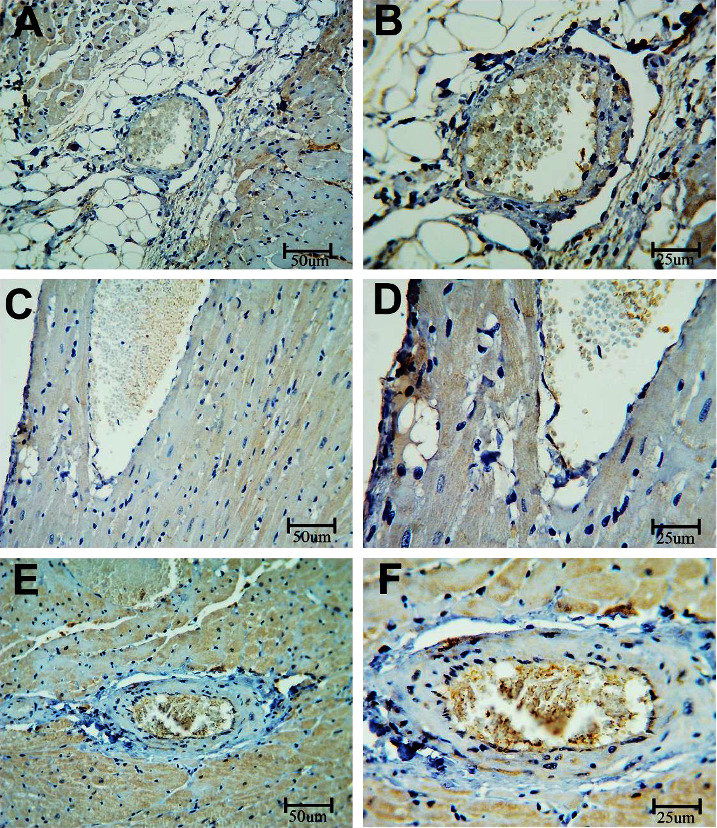
Histopathological section in the coronary artery of rat stained with eNOS immune stain. a and b are the control group, c and d are the BPA group, and e and f are the BPA + LC group. The coronary artery of the control group shows a minimal expression of the dye in the wall of the artery. The expression of the color is notably increased in the BPA group and reduced in the BPA + LC group (eNOS immune stain; A, C, and E X 200; B, D, and F X 400).

**Figure 10:**
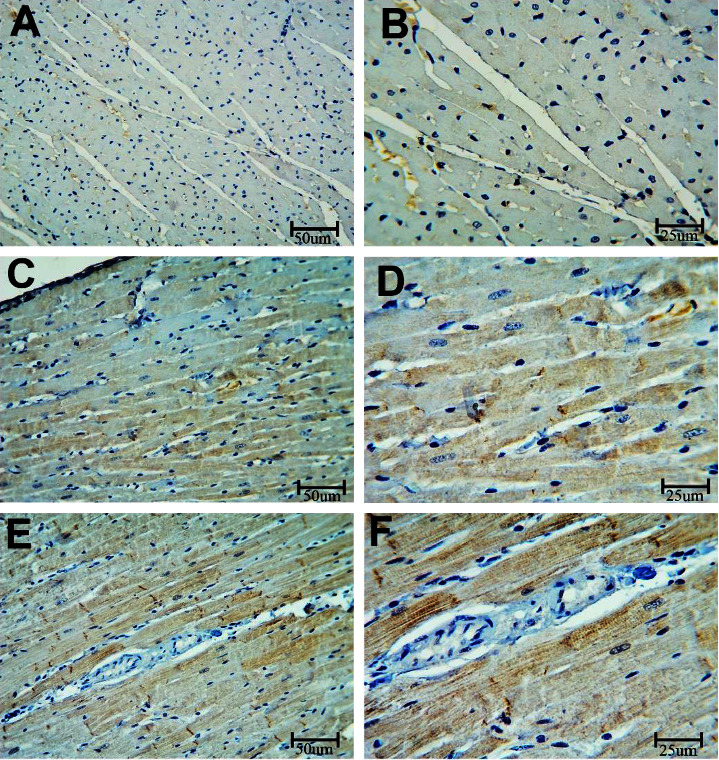
Histopathological section in the heart of rat stained with eNOS immune stain. a and b are the control group, c and d are the BPA group, and e and f are the BPA + LC group. The cardiac muscle of the control group shows a minimal expression of the dye in the wall of the artery. The expression of the color is notably increased in the BPA group and reduced in the BPA + LC group (eNOS immune stain; A, C, and E X 200; B, D, and F X 400).

**Figure 11:**
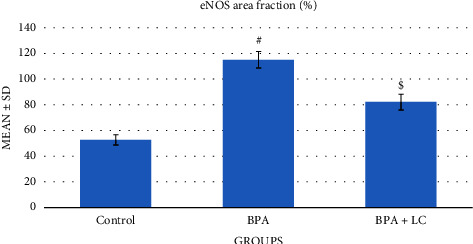
Graph demonstrating the quantitative analysis of eNOS expression area percentage of the heart. Data are expressed as means ± SDs. ^#^: significantly different from the control group, while ^$^significantly different from the BPA group (*P* < 0.001).

In our study, the endothelial function was assessed by staining coronary artery sections with the eNOS immune stain (Figure 9). BPA caused an enhanced expression of eNOS when compared with the control group (*P* < 0.001). Conversely, coadministration of LC caused a significant reduction in eNOS expression in the coronary artery (*P* < 0.001) (Table 1). Regarding the myocardium, Figure 10 demonstrated that the cardiac muscle showed an increased expression of the eNOS in rats exposed to the BPA group (*P* < 0.001). On the other hand, there was a decreased expression of the stain in the BPA + LC group (*P* < 0.001). The quantitative analysis of the eNOS expression area % of the heart (Figure 11) revealed increased eNOS expression in BPA-treated rats in comparison with control rats (114.84 ± 6.21% versus 52.39 ± 4.05%). However, coadministration of LC and BPA was associated with a significant decrease in eNOS expression in comparison to the BPA group (81.86 ± 5.94% versus 114.84 ± 6.21%) (*P* < 0.001).

### 3.3. Tissue Glutathione and Malondialdehyde

The statistical analysis of tissue MDA and GSH demonstrated a highly statistically significant difference (*P* < 0.001) between BPA-treated rats and control rats (approximately 3-fold for MDA and 2.5-fold for GSH). In the group of BPA + LC, the MDA demonstrated a statistically significant decrease (*P* < 0.001) of the mean MDA level (20.75 nmol/g versus 32.82 nmol/g) while the GSH demonstrated a statistically significant rise (*P* < 0.001) of mean GSH level (1.22 mmol/g versus 0.82 mmol/g) (Table 2).

## 4. Discussion

BPA is a pollutant that induces different health problems including metabolic disorders, hormonal-based tumors, and cardiovascular diseases [34]. According to Melzer et al. [35], humans with greater urinary BPA concentrations seemed to be more susceptible to heart disease. Data do exist about BPA's toxic effects on the heart; however, there is little data regarding such an effect on the coronary artery. Hence, in this research, BPA's effects on the heart and coronary artery were investigated. A BPA dose of 40 mg/kg/d was used in our study. This dose was selected from recently published studies that used a BPA dose of 40 mg/kg/d [36, 37].

The current study demonstrated significant histopathological and immunohistochemical changes in the coronary arteries and the hearts of the rats. Starting with coronary artery changes, the current BPA-associated histopathological alterations in the coronary artery, which included vacuolation of cells in the media and detachment of the endothelium of the intima of the artery, and increased collagen fibers in the arterial wall, are suggestive of coronary artery vasculitis and atherosclerosis. These changes can be elucidated by the influence of BPA on the endothelial cells, resulting in elevated levels of surface adhesion molecules that increase permeability and induce immune cell infiltration [38]. BPA could also enhance the inflammatory response by increasing the serum levels of proinflammatory cytokines, ending by apoptosis of coronary artery smooth muscle cells [38], which was also evident in our study by immunohistochemistry. Moreover, BPA-induced endothelial cell changes could lead to coronary vascular weakness and ventricular hemorrhage, which appear as cardiac dysfunction [39]. Melzer et al. [40] stated that BPA exposure was linked to an enhanced risk of CAD. The latter has been suggested to be related to BPA by different receptor-mediated toxicity such as estrogenic and antiandrogenic receptors, which finally affect the cardiovascular tissues.

On the other hand, the myocardium of BPA-exposed rats showed congestion of the blood vessels and infiltration by polynuclear cells in the myocardium. These findings are suggestive of the inflammation linked to BPA administration. Proinflammatory cytokines such as TNF-*α* and IL-1*β* can reduce the left ventricular function and cause loss of cardiomyocytes via apoptosis. These cytokines also have the ability to induce neurohumoral activation and cause oxidative damage, resulting in the initiation of the p38-MAP kinase and nuclear factor-*κ*B. Collectively, this induces cardiomyocytolysis and reduces Ca^+2^ uptake by the sarcoplasmic reticulum, and therefore impairs cardiac inotropy [16]. The congested and dilated blood vessels could explain the enhanced cellular infiltrates in-between muscle fibers in the BPA-treated group. Such findings are consistent with Klint and coworkers [41], who reported that BPA increased vascular endothelial growth factor and eNOS that controls vascular tone in cardiac tissues.

In addition, the myocardial changes were suggestive of increased fibrosis as indicated by a significant increase in collagen fibers. Similar findings in the myocardium have been reported previously after oral administration of BPA (50 mg/kg) to rats, where the myocardium of BPA-exposed rats showed a significantly increased collagen fiber deposition in comparison to control rats [16]. Also, Bahey et al. [42] reported that BPA-exposed animals showed structural myocardial abnormalities, which included disarrangement of myofibers, hypertrophy of myocytes, and fibrosis. Myocardial fibrosis was reported previously as well after the exposure of rats to a higher BPA dose [43]. It commonly occurs in many cardiac diseases including heart infarction, failure, and hypertension [44]. BPA-induced myocardial fibrosis is mostly promoted by the cardiac fibroblasts' proliferation, collagen production, and mast cell activation [45].

In our work, the apoptosis caused by BPA was evaluated by the immunohistochemical study of the proapoptotic marker caspase-3. BPA caused apoptosis in the coronary artery and the cardiac tissue as indicated by enhanced caspase-3 expressions. The immunohistochemistry examination of caspase-3 in cardiac slices revealed that caspase-3 expression was minimal in the control group, while it is significantly elevated in the BPA group and decreased in the BPA + LC group. Indeed, treatment of rats with LC improved the apoptotic effects of BPA, as evidenced by down-regulatingcaspase-3 expressions. Myocyte apoptosis is associated with high concentrations of proinflammatory cytokines including IL-1*β*, NF-*α*, and interferon-*γ* [46].

Due to the importance of eNOS activity to keep the vascular dilatation, eNOS expression was investigated in this study. Nitric oxide (NO) is produced by the endothelial nitric oxide synthase (eNOS) enzyme by converting the L-arginine to NO in the vasculature [47]. The eNOS is expressed in endocardial cells, endothelial cells, myocytes, and other myocardial cells [48]. NO underlies smooth muscles and acts as an endothelium-dependent vasodilator [49]. The reduced NO level results in vasoconstriction, endothelial dysfunction, and hypertension [50]. Also, it was revealed that a deficiency of eNOS accelerated atherosclerotic lesion formation in eNOS knockout mice [51].

Our findings revealed an increased expression of eNOS in BPA-exposed rats, which was in agreement with Klint et al. [41]. It should be mentioned that this finding is not against the previously mentioned concept that eNOS deficiency is associated with atherogenesis. To clarify, eNOS may have two faces in the pathophysiology of atherosclerosis. This depends on the vascular tissue levels of tetrahydrobiopterin. For example, eNOS overexpression may promote atherogenesis through increased free radical production from dysfunctional eNOS, i.e., eNOS-mediated superoxide generation [51]. Interestingly and supporting our results, it was postulated that oxidative stress (which was evident to be induced by BPA in our study) can enhance the eNOS expression, and, indeed, eNOS expression is enhanced in most types of vascular disease. This might be an attempt of the organism to compensate for the decreased NO activity. However, such compensation is usually ineffective, as the eNOS becomes or remains uncoupled under pathologic conditions. The upregulation of eNOS expression makes the condition even worse as the uncoupled eNOS worsens oxidative damage [48]. Moreover, other different mechanisms could modulate the BPA-induced eNOS expression including the Ca^2+^/calmodulin-dependent mechanism [52], and the angiotensin II pathway [53].

At the biochemical level, BPA-induced oxidative stress was investigated. The biochemical analysis showed the occurrence of oxidative damage in BPA-treated rats that was indicated by the reduced level of the antioxidant GSH and the elevated level of the lipid peroxidation marker (MDA). The heart is significantly sensitive to free radicals due to its high oxidative metabolism and due to its few antioxidant defenses. ROS are cytotoxic agents, which cause oxidative stress by targeting biomolecules like membrane lipids and cellular DNA [16]. Therefore, oxidative stress could be the cause of the structural alterations observed in the hearts of BPA-treated rats. Oxidative stress could also trigger cardiac fibrosis via different mechanisms, such as stimulation of transforming growth factor-beta1 expression [54] and the JAK/STAT signaling pathway [55]. Thus, the current data may indicate a possible correlation between BPA-mediated oxidative stress and cardiac fibrosis. Other studies have reported BPA-induced oxidative damage in the myocardial tissue of adult male albino rats. For instance, the study by Abd El-Haleem and Abass [16] revealed a significant rise in serum MDA and a significant reduction in tissue-reduced GSH and catalase in the myocardium of BPA-treated animals. Similarly, it was found that BPA caused a significant rise in MDA, and a significantly decreased catalase [21].

The increased evidence of BPA-related negative effects on health has encouraged researchers to search for a drug or natural substance that protects against these effects, especially for those at high exposure risk [42]. This study reports, for the first time, that LC has a significant protective effect against BPA-related coronary and myocardial toxicity. It was clear that LC could reduce the BPA-induced lipid peroxidation and elevate the antioxidant GSH levels as well. Similarly, LC showed protective effects against isoproterenol-induced myocardial infarction by reducing oxidative damage markers and inflammatory cell infiltration [56]. In our study, the BPA-induced histopathological alterations were reduced in the LC-treated group, which was indicated by the lower cells' infiltration, normal myocardial fiber structure, and normal coronary artery architecture. These findings are explaining the ability of LC to reduce cardiac fibrosis pathogenesis and myocardial infarction, which are consistent with many previous reports [56–58]. Moreover; LC could attenuate the BPA-induced eNOS expression revealing its protective effect against the probable PBA-induced coronary artery vasoconstriction. It is known that the LC role is enhanced by different mechanisms, such as calcium channel regulation, endothelial integrity maintenance, and cellular homeostasis control [59]. Thus, LC can protect against the myocardial and coronary artery damage that commonly occurs in cardiovascular diseases.

## 5. Conclusion

Exposure to BPA has been reported to be linked to CVD. However, few studies have focused on its association with coronary artery pathology. This study concluded that BPA exposure can result in biochemical and pathologic alterations in the myocardium and coronary artery. The results suggest that oxidative stress, fibrosis, and apoptosis play important roles in BPA-related toxicity. Importantly, the harmful alterations caused by BPA could be partially improved by the coadministration of LC. However, more studies are required to evaluate the effects of prolonged exposure to different BPA doses on the cardiovascular system. Also, it is recommended to regularly administer LC to help reduce the deleterious effects of exposure to BPA-containing products.

## Figures and Tables

**Table 1:** Comparison between the study groups regarding Masson trichrome area fraction (%), caspase area fraction (%), and eNOS area fraction (%).

	Control group	BPA group	BPA + LC	Test of significance	Within group significance
Masson trichrome area fraction (%)	11.11 ± 1.54	47.34 ± 3.60	30.02 ± 4.09	*F* = 259.593	*P*1 < 0.001^*∗*^
				*P*=0.001^*∗*^	*P*2 < 0.001^*∗*^
					*P*3 < 0.001^*∗*^

Caspase area fraction (%)	182.92 ± 6.44	240.69 ± 4.84	204.47 ± 10.59	*F* = 115.535	*P*1 < 0.001^*∗*^
				*P* < 0.001^*∗*^	*P*2 < 0.001^*∗*^
					*P*3 < 0.001^*∗*^

eNOS area fraction (%)	52.39 ± 4.05	114.84 ± 6.21	81.86 ± 5.94	*F* = 245.417	*P*1 < 0.001^*∗*^
				*P* < 0.001^*∗*^	*P*2 < 0.001^*∗*^
					*P*3 < 0.001^*∗*^

*F*: one-way ANOVA test, *p*1: difference between control and BPA groups, *P*2: difference between control and BPA + LC groups, *P*3: difference between BPA group and BPA + LC groups. Data are presented as means ± SDs. ^*∗*^indicates a statistically significant difference.

**Table 2:** Comparison between the study groups regarding MDA and GSH.

	Control group	BPA group	BPA + LC	Test of significance	Within group significance
MDA (nmol/gram tissue)	11.53 ± 0.98	32.82 ± 2.53	20.75 ± 2.60	*F* = 193.365	*P*1 < 0.001^*∗*^
				*P* < 0.001^*∗*^	*P*2 < 0.001^*∗*^
					*P*3 < 0.001^*∗*^

GSH (mmol/gram tissue)	1.95 ± 0.13	0.82 ± 0.10	1.22 ± 0.19	*F* = 119.995	*P*1 < 0.001^*∗*^
				*P*=0.001^*∗*^	*P*2 < 0.001^*∗*^
					*P*3 < 0.001^*∗*^

*F*: one-way ANOVA test, *p*1: difference between control and BPA groups, *P*2: difference between control and BPA + LC groups, *P*3: difference between BPA group and BPA + LC groups. Data are presented as means ± SDs. ^*∗*^indicates a statistically significant difference.

## Data Availability

All data used to support the findings of the current study are included within the article.
